# Optical Coherence Tomography to Assess Proximal Side Optimization Technique in Crush Stenting

**DOI:** 10.3389/fcvm.2022.861129

**Published:** 2022-03-15

**Authors:** Francesco Lavarra, Giuseppe Tarantini, Davide Sala, Vasile Sirbu

**Affiliations:** ^1^Cardiovascular Department, Jilin Heart Hospital, Changchun, China; ^2^Interventional Cardiology Unit, Department of Cardiac, Thoracic and Vascular Sciences, University of Padua Medical School, Padua, Italy

**Keywords:** bifurcation lesions of coronary arteries, Optical Coherence Tomography, percutaneous coronary interventions (PCI), crush stenting technique, stent optimization

## Abstract

**Aim:**

The aim of this study was to explore the potential intraprocedural benefits of the Proximal Side Optimization (PSO) technique by Optical Coherence Tomography (OCT).

**Methods:**

A case series of 10 consecutive true bifurcation lesions, with severe long pathology of long side branch (SB), were randomly assigned to be treated by standard DK Crush procedure (non-PSO group) as compared to DK Crush in PSO modification (PSO group). The data from OCT investigation before crushing of the SB Drug-Eluting Stent (DES), after crushing, after first kissing balloon inflation (KBI), and after final angiography were compared between the two groups (Public trials registry ISRCTN23355755).

**Results:**

All 10 cases were successfully treated by the assigned technique. The two groups were similar in terms of indications for the procedure, bifurcation angle, and stent dimensions. As compared to the non-PSO, the PSO group showed larger proximal SB stent areas (5.8 ± 1.8 vs. 4.5 ± 0.5 mm^2^; *p* = 0.02), the larger delta between distal and proximal stent areas before crush (1.5 ± 0.7 vs. 0.6 ± 0.5 mm^2^; *p* = 0.004), and the larger Space of Optimal Wiring (SOW) after Crush (5.3 ± 1.8 vs. 2.5 ± 1.1 mm^2^; *p* = 0.02). The gaps in scaffolding within the ostial segment of the Side Branch DES were found in two patients from the non-PSO group.

**Conclusion:**

The DK Crush in PSO modification results in larger SB DES and SOW areas with better apposition to the vessel wall. As result, the SB DES acquires a funnel shape, which reduces the risk of passage outside the SB stent struts during re-wiring, thus, allowing predictable and secure results.

## Introduction

The Proximal Side Optimization (PSO) is the last proposed technique adjustment of DK crush, based on a Side Branch (SB) stent post-dilatation prior to the crush (1–3), Supplementary Material. Given the high resolution and the capability for three-dimensional (3D) reconstruction, we sought to use Optical Coherence Tomography (OCT) to further explore the potential intraprocedural benefits of this modification in a series of patients treated by two-stent DK Crush approach for true bifurcation lesions.

## Methods

**Figure 1:**
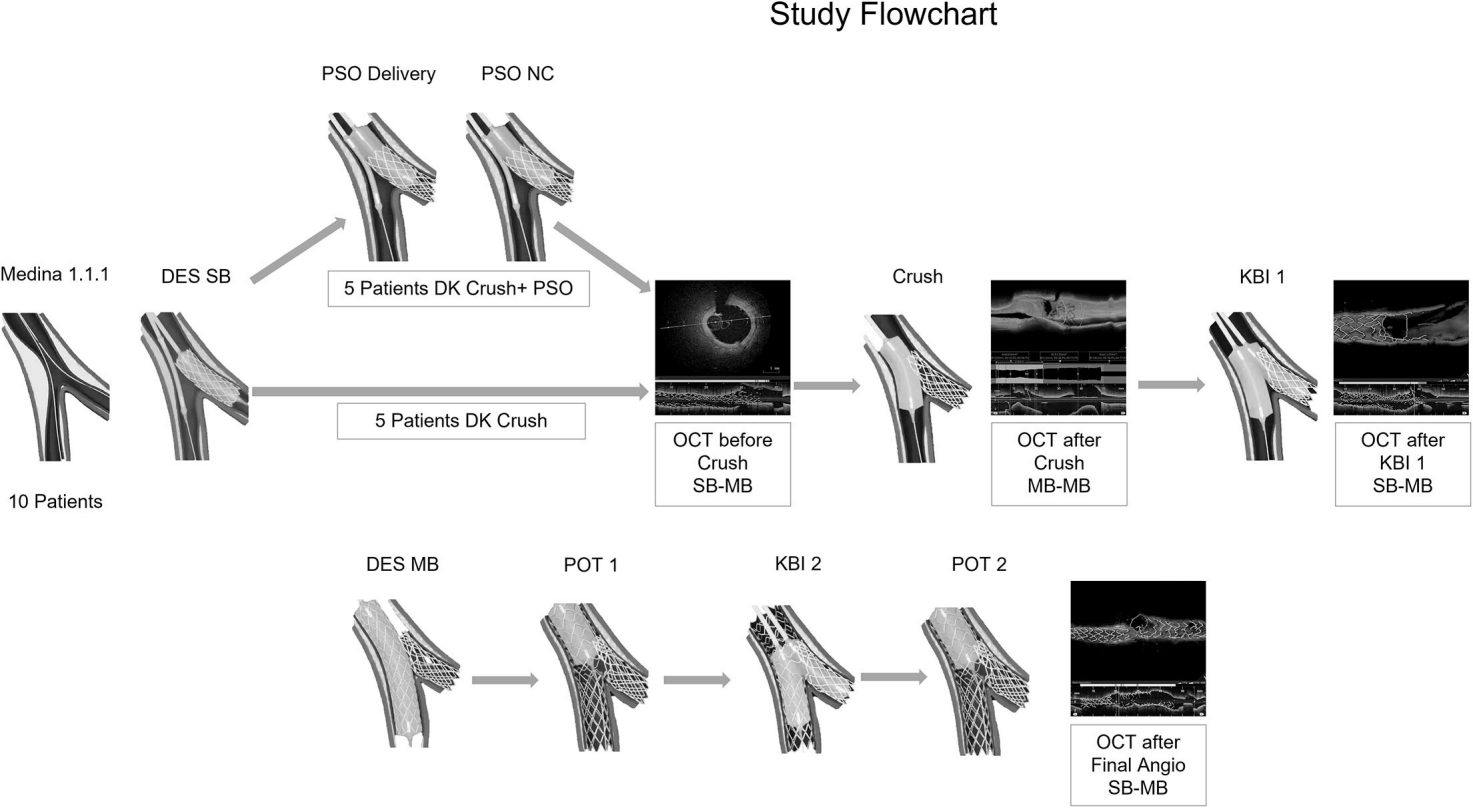
Case series flowchart. Ten bifurcation lesions were randomly assigned in a 1:1 ratio to either double kissing (DK) crush or DK Crush in proximal side optimization (PSO) modification. The procedures were conducted by angiography guidance. Optical Coherence Tomography (OCT) examinations were performed in all patients for 4 times: before Crush, after Crush, after Kissing Balloon Inflation 1, and after final angiography. The OCT findings were analyzed offline, after the end of the procedure.

**Figure 2:**
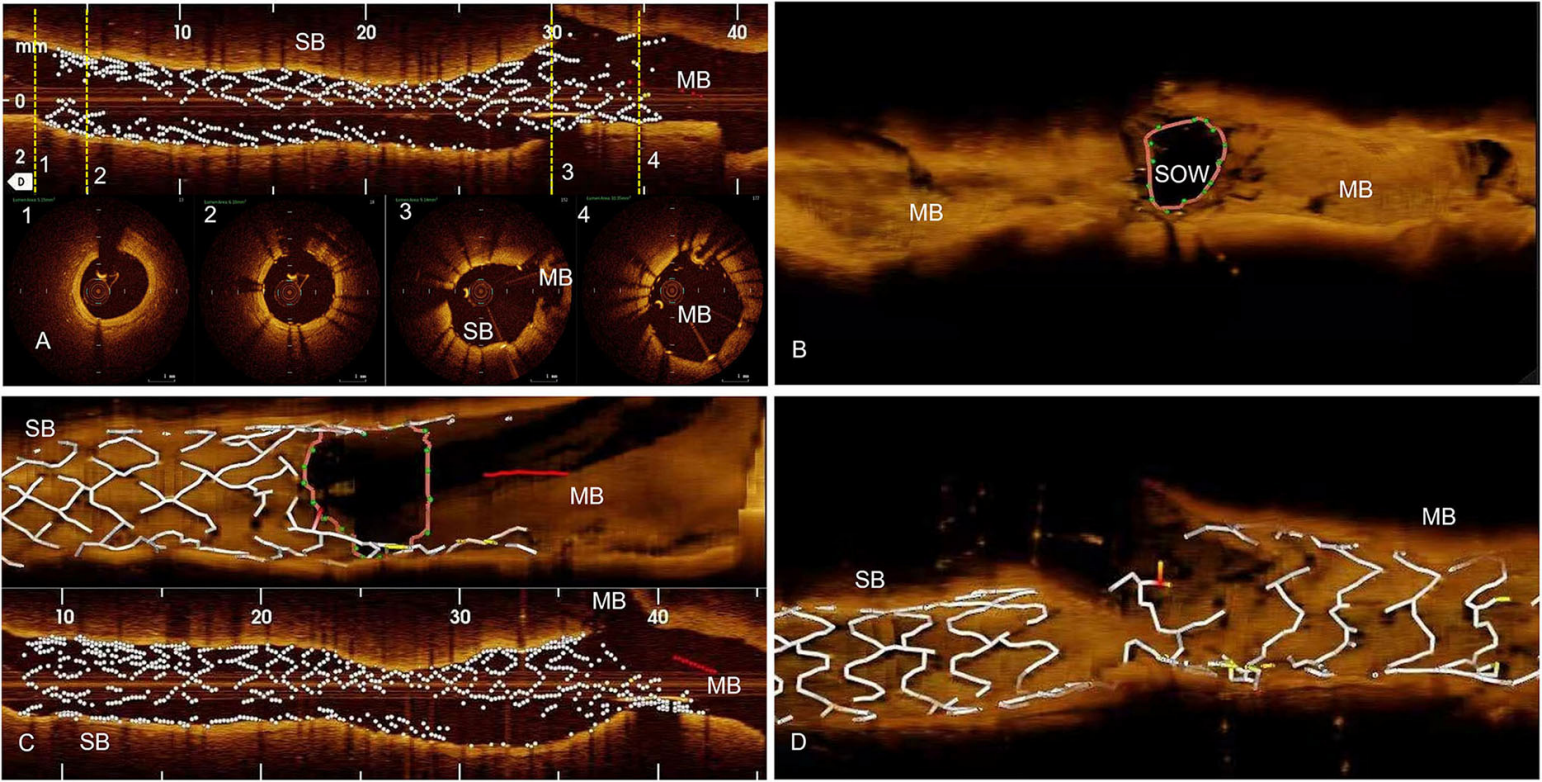
Methods, OCT examinations. **(A)** The first OCT pullback is done from Side Branch (SB) toward Main Branch (MB), before SB Drug Eluting Stent (DES) is crushed. We analyze distal reference segment (1), distal DES (2), ostial DES (3), and the protruding in MB DES (4) areas. **(B)** The second OCT pullback is done in the MB after the SB DES is being crushed. Using the 3D reconstruction, we analyze the area of Space of Optimal Wiring (SOW). **(C)** Third OCT pullback is done from SB to MB after Kissing Balloon one (KBI1) and we analyze for possible SB DES distortions in longitudinal reconstructions, and SB DES ostial area in 3D reconstruction. **(D)** The fourth OCT pullback after final angiography, from SB to MB, to assess for possible gaps in stent coverage in the polygon of confluence.

Ten bifurcation lesions, all Left Anterior Descending Artery, Diagonal Branch Bifurcation (LAD-D) Medina 1.1.1, with long (>10 mm) and severe stenoses (>75%) of the long (>75 mm) SB, were randomly assigned in a 1:1 ratio to either Double Kissing Crush bifurcation stenting (DK Crush) of the non-PSO group (4), or DK Crush in PSO modification of the PSO group (1). All PCI procedures were angiography-guided and all lesions were treated using Xience V (Abbott Vascular, CA, USA) DES. All SB DES were positioned with an adequate protrusion in Main Branch (MB) (3–5 mm depending on bifurcation angle) and deployed at nominal pressure. Five patients had further PSO modification (PSO group), which briefly consists in post-dilatation of the SB DES with the delivery balloon pulled back halfway in the MB at a nominal + 6 atm pressure, followed by a larger 0.5-mm non-compliant balloon prior to the crush step (Figure 1). The first and second KBIs were performed with the non-compliant balloons intended for POT in MB and 0.5 mm larger-than-stent diameter in SB in all patients. Sequential high-pressure dilatations before KBI were performed in all patients. The first and second POTs were used for all MB stents in all patients. The OCT pullbacks were performed in all patients 4 times during the PCI procedure using a commercially available FD-OCT system (Cornaris™, Intravascular Imaging System, Clear View, Shenzhen, China). The first pullback from SB to MB was done before crushing the SB stent; the second pullback was performed in the MB from distal to proximal segment, after crushing the SB stent; the third pullback was performed after the first KBI from SB to MB; the fourth pullback was performed from SB to MB, after final angiography (Figure 2). The rationale for the first pullback was: (1) to assess the distal reference segment in terms of diameters and areas; (2) to assess distal stent segment expansion in terms of diameters and areas as compared to distal reference segment; (3) to assess the ostial segment of the SB DES in terms of expansion and area as compared to distal DES segment; (4) to assess the diameters and areas of the protruding into the MB SB DES as compared to its ostial segment (Figure 2A). The rationale for the second pullback was to assess the Space of Optimal Wiring (SOW) after the SB DES is being crushed in terms of area, using the 3D reconstruction (Figure 2B). The rationale of the third pullback was to assess the SB stent expansion and to analyze for possible stent distortions following the KBI 1 (Figure 2C). The fourth pullback was done after final angiography to assess for possible gaps in the polygon of confluence (Figure 2D). An automated contrast delivery system (ACIST Medical Systems, Eden Prairie, MN) was used for contrast delivery through the guide catheter at a rate of 4 ml/s, zero rate of rise, and a pressure of 400 psi. An average of 14 ml of contrast (Visipaque GE Healthcare, Piscataway, NJ, USA) was used in each run. Images were calibrated by an automated adjustment of the Z-offset, and an automated pullback was set at 20 mm/s for 3 s. The hemodynamic and electrocardiogram changes were monitored during each injection of contrast for image acquisition. The adequate hydration protocols were applied to all study patients pre- and post-procedure, and the kidney function was monitored for eventual Contrast-Induced Nephropathy. The OCT analysis was performed at the end of the PCI procedure. All the measurements were performed automatically by the OCT software after thorough calibration and elimination of the eventual artifacts, thus, excluding the bias of human factors in results. Categorical variables are presented as numbers and frequencies and were compared by the Fisher exact test. The continuous variables analyzed for normality of distribution with Shapiro–Wilk test (resulting in *p* > 0.05) are expressed as mean ± SD and were compared by the Mann–Whitney *U* test, given the small sample size. All analyses were performed with SAS statistical software (version 9.1, SAS Institute, Inc., Cary, North Carolina). The study was approved by the Institutional Committee on Human Research at the Jilin Heart Hospital (Hospital Ethics Committee approval R 15/21-JHH45 from January 15, 2021: public trials registry ISRCTN23355755), and all the participants signed the informed consent for participation in the study.

## Result

**Figure 3:**
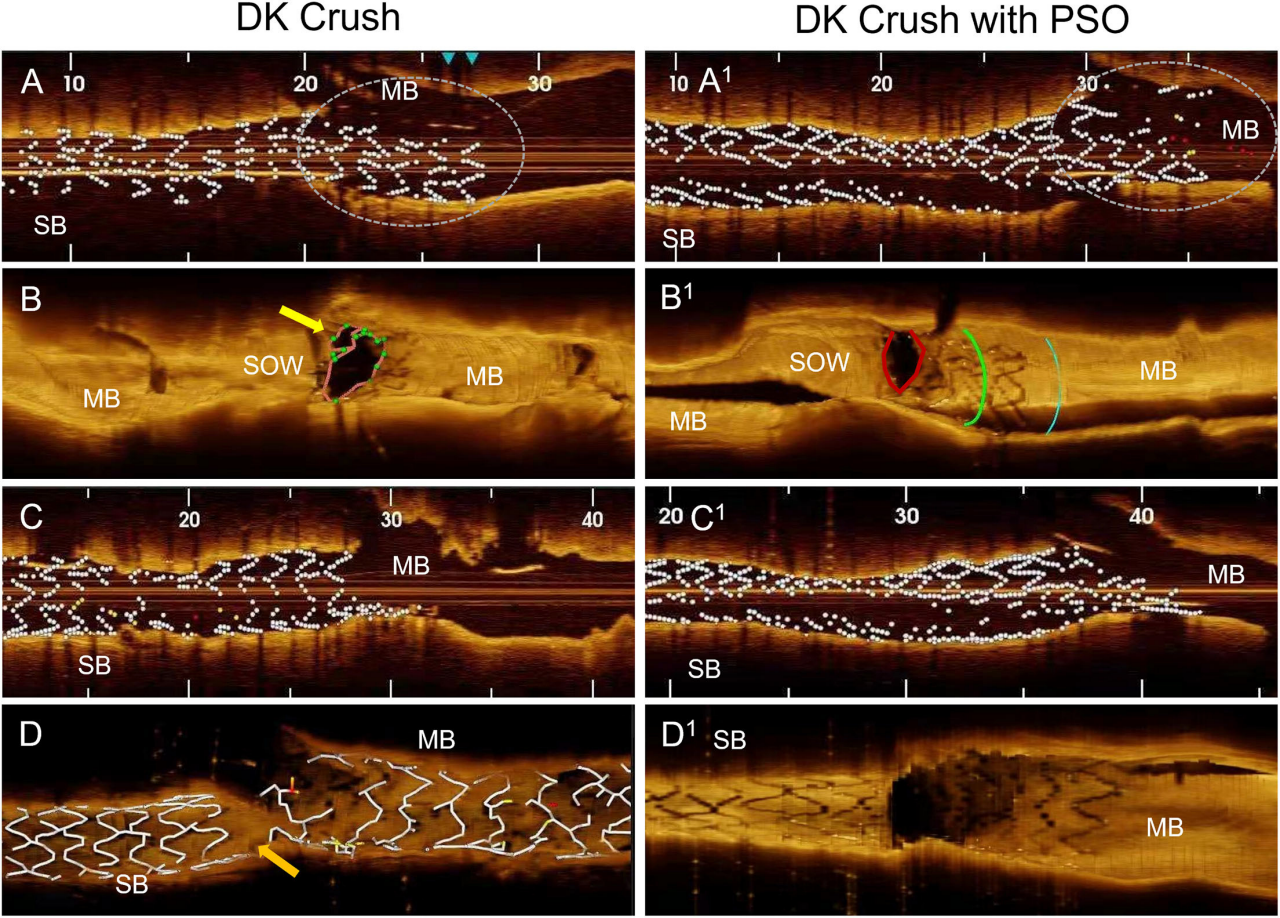
OCT findings in DK Crush compared to DK Crush in PSO modification. **(A)** After DES implantation on SB at nominal pressure, the OCT shows equal proximal and distal stent diameters, the protruding stent segment in the main branch is under-expanded (circle). **(B)** After crushing, the SB DES reveals a space between the stent and the ostium of the side branch for inadequate wire passage (yellow arrow). **(C)** After the first KBI, the stent has tubular shape. **(D)** After final angiography, depicting a gap in stent coverage in the polygon of confluence (orange arrow). **(A**^**1**^**)** After DES implantation on SB and PSO modification, shows larger proximal stent diameter compared to distal segment, full apposition of the protruding stent segment into the main branch (circle). **(B**^**1**^**)** After crushing the SB DES, the optimal apposition to the side branch ostium with no space for inadequate wiring. **(C**^**1**^**)** After the first kissing balloon inflation, reveals a funnel like shape of the stent. **(D**^**1**^**)** After final angiography, complete coverage by stents of the polygon of confluence. MB, Main Branch; SB, Side Branch; SOW, Space of Optimal Wiring; PSO, Proximal Side Optimization.

All 10 cases were successfully treated by the assigned technique. The two groups had similar indications, bifurcation angles, and stent size. As compared to the non-PSO, the PSO group showed larger DES areas (5.8 ± 1.8 vs. 4.5 ± 0.5 mm^2^; *p* = 0.02), and a larger increase in areas in the ostial segment (1.5 ± 0.7 vs.0.6 ± 0.5 mm^2^; *p* = 0.004) and the protruding in MB segment as compared to the distal segment of the DES (7.8 ± 3 vs. 4.8 ± 1 mm^2^; *p* = 0.04) before Crush (Table 1, Figure 3A). The SOW areas after Crush were significantly larger in the PSO group [(5.3 ± 1.8 vs. 2.5 ± 1.1 mm^2^; *p* = 0.02)] (Figures 3B,C). The gaps in scaffolding within the ostial segment of the SB DES were found in two patients from the non-PSO group (Figure 3D).

**Table 1:** Procedural characteristics and Optical Coherence Tomography (OCT) Findings.

**Variables**	**Overall 10 patients**	**PSO 5 patients**	**non-PSO 5 patients**	***P*-Value**
Age	63.3 ± 7	63 ± 7	64 ± 6	0.90
Male gender, n (%)	5 (50)	2 (40)	3 (60)	1.00
Unstable Angina, n (%)	10 (100)	5 (100)	5 (100)	1.00
Bifurcation angulation, °	47.0 ± 5.4	47.2 ± 6.2	46.7 ± 4.7	0.89
SB DES diameter, mm	2.6 ± 0.1	2.6 ± 0.3	2.6 ± 0.1	0.60
SB DES length, mm	33.0 ± 9.9	32.5 ± 4.4	34.0 ± 18.3	0.84
MB DES diameter, mm	2.9 ± 0.6	3.4 ± 0.5	3.3 ± 0.7	0.86
MB POT balloon diameter, mm	3.7 ± 0.6	3.8 ± 0.7	3.5 ± 0.5	0.60
SB KBI balloon diameter, mm	3.1 ± 0.5	3.0 ± 0.5	3.1 ± 0.6	0.80
SB distal reference segment area, mm^2^	3.6 ± 1.2	3.6 ± 1.3	3.7 ± 0.4	1.00
SB distal DES segment area, mm^2^	4.3 ± 1.4	4.4 ± 1.6	4.1 ± 0.1	0.93
SB ostial DES segment area, mm^2^	5.4 ± 1.8	5.8 ± 1.8	4.5 ± 0.5	0.02
SB DES protruding in MB area, mm^2^	6.9 ± 2.8	7.8 ± 3.0	4.8 ± 1.0	0.04
SB DES ostial vs. distal segment delta in area, mm^2^	1.1 ± 0.8	1.5 ± 0.7	0.6 ± 0.5	0.004
% increase in DES area in ostial segment	27.9 ± 22.3	37.3 ± 21.1	9.3 ± 9.5	0.05
SOW area, mm^2^	4.5 ± 2.2	5.3 ± 1.8	2.5 ± 1.1	0.02
Gaps in vessel coverage n (%)	2 (20)	0 (0)	2 (40)	0.06

*SB, Side Branch; DES, Drug Eluting Stent; MB, Main Branch; SOW, Space of Optimal Wiring*.

## Discussion

Since its first presentation, the Crush technique has had several adjustments (5). A 2-step post-dilation involving a high-pressure post-dilation in the SB, followed by final KB, significantly reduced SB ostial stenosis (6). The high-pressure NC balloon inflation at the ostium of the SB stent after Crush and prior to MB stent implantation aimed to facilitate the SB access after MB stenting (7). The POT aimed to facilitate access to the SB after KBI (8). Finally, the DK Crush modification demonstrated higher rates of final KBIs and resulted in better clinical outcomes (4, 9, 10). The post-dilatation of the implanted stent is a universally recognized step during the PCI procedure. Moreover, it is an important step in the DK Crush procedure itself, the stent implantation in the main branch is followed by POT 1 before any further manipulations on the stent. All the previously described modifications of this technique were advocated in the post dilatation of the SB only after the stent crushing.

To note, considering that stent size is based on distal vessel segment diameter, there is a concrete risk in deploying the stent proximally to avoid distal dissection. This malposition leaves room for inappropriate wire track under the stent struts during rewiring as demonstrated previously in bench models (6). In the current study, we noticed, by using OCT, that this phenomenon can occur while performing the DK Crush procedure without PSO modification in *in vivo* interventions (Figure 3). Our modification advocates the SB stent post-dilatation in its proximal segment immediately after deployment, using a delivery balloon and a.5 mm larger diameter NC balloon. This results in adequate stent expansion and apposition before it is crushed, eliminating the residual space between stent and vessel wall and the risk for inadequate wire passage during rewiring. Moreover, the acquired funnel shape of the SB stent is anchoring it in the ostial position, thus, preventing its dislodgement downstream, due to subsequent procedural steps (Figure 3C). A recent paper presented a detailed step-by-step description of troubleshooting for each stage of the DK crush technique. The gaps in stent coverage of the carina were attributed to the very distal cell rewiring (11). In our case series, this modification allowed us to avoid this drawback, independent of the crossed cell, due to the above-mentioned mechanisms.

## Conclusion

The DK Crush in PSO modification results in larger SB DES and SOW areas with better apposition to the vessel wall. As a result, the SB DES acquires a funnel shape, which eliminates the risk of passage outside the SB stent struts during re-wiring, thus, allowing predictable and secure results.

## Data Availability Statement

The original contributions presented in the study are included in the article/Supplementary Material, further inquiries can be directed to the corresponding author.

## Ethics Statement

The studies involving human participants were reviewed and approved by Jilin Heart Hospital. The patients/participants provided their written informed consent to participate in this study.

## Author Contributions

FL, GT, and VS contributed to the conception and design of the study. DS organized the database and performed the statistical analysis. VS wrote the first draft of the manuscript. FL, GT, DS, and VS wrote sections of the manuscript. All authors contributed to manuscript revision, read, and approved the submitted version.

## Conflict of Interest

The authors declare that the research was conducted in the absence of any commercial or financial relationships that could be construed as a potential conflict of interest.

## Publisher's Note

All claims expressed in this article are solely those of the authors and do not necessarily represent those of their affiliated organizations, or those of the publisher, the editors and the reviewers. Any product that may be evaluated in this article, or claim that may be made by its manufacturer, is not guaranteed or endorsed by the publisher.

## Abbreviations

PCI: Percutaneous Coronary Interventions
DK Crush: Double Kissing Crush bifurcation stenting
KBI: Kissing Balloon Inflation
PSO: Proximal Side Optimization
POT: Proximal Optimization Technique
OCT: Optical Coherence Tomography
SOW: Space of Optimal Wiring
MB: Main Branch
SB: Side Branch.
