# Supplementary material for: Optical Coherence Tomography to Assess Proximal Side Optimization Technique in Crush Stenting
Source: Front Cardiovasc Med. 2022 Mar 15;9:861129. doi: 10.3389/fcvm.2022.861129 (PMC8965003; doi:10.3389/fcvm.2022.861129)

Supplement

Proximal Side Optimization in Crush Stenting

DK Crush procedure is an established treatment algorithm of coronary artery bifurcation lesions with proven efficacy in multiple randomized clinical studies [1]. The first step in this algorithm is stent implantation in the Side Branch (SB). The deployment of the SB stent at nominal pressure bears the risk of not achieving the perfect expansion when there is important difference between proximal and distal diameter of side branch. Coronary wire tracking in the free space between vessel wall and stent can cause subsequent SB stent distortion by the following steps of the algorithm (Figure 1) [2]. This phenomenon was observed by Ormiston, using micro-computed tomographic imaging of bench Crush procedure. The study demonstrated the risk of gaps in stent scaffolding on the side of the SB stent opposite to the crushed segment (Figure 2) [3]. In order to avoid this phenomenon, DK Crush Investigators recommended guiding wire tracking in the most proximal cell of the crushed stent [1], a maneuver which cannot be performed with perfect precision in everyday practice under fluoroscopic guidance.

The Proximal Side Optimization (PSO) is the last proposed technique adjustment of DK crush, based on a SB stent post-dilatation prior to the crush. It consists in post dilatation of the deployed SB stent by delivery balloon that is retrieved half-way back in the Main Branch (MB) followed by post dilatation with High Pressure balloon across the bifurcation before crushing (Figure3) [4]. Using silicone bench model, we demonstrated that PSO improves stent expansion, increases the Space of Optimal Wiring (the space of correct wire tracking towards the SB stent lumen instead of the space between stent cells and vessel wall) (Figure 4) [5].

Contribution to the field. In the actual paper, we used in-vivo intracoronary imaging findings during the DK Crush procedure to further demonstrate the rationale and benefits of this modification. A wider acceptance by the interventional community of this simple modification could help in anticipating the drawbacks and avoid the need in troubleshooting during Crush stenting in bifurcation lesions.

References

1. Zhang JJ, Chen SL. Classic crush and DK crush stenting techniques. EuroIntervention. 2015;11:V102-5.
2. Lavarra F, Sala D, Sirbu V. Proximal side optimization in crush stenting. A step-by-step technical approach. Romanian Journal of Cardiology. 2020;3:382-8. DOI:10.47803/rjc.2020.30.3.382.
3. Ormiston JA, Webster MWI, Webber B, et al. The "crush" technique for coronary artery bifurcation stenting: insights from micro-computed tomographic imaging of bench deployments JACC Cardiovasc Interv. 2008;1:351-7.
4. Lavarra F. Proximal Side Optimization: A modification of the double kissing crush technique. US Cardiology Review 2020;14:e02.
5. Lavarra F. Proximal Side-Branch Optimization in Crush stenting. A step-by-step Technical Approach in a Silicone Phantom Model. Cardiovascular Revascularization Medicine. 2020;S1553-8389;30464-4.

Figure Legends

Figure 1. Graphic representation of the risks of Side Branch (SB) stent Crush in unpredictable fashion and subsequent guiding wire crossing under stent struts. A- SB stent deployed at nominal pressure results in under expansion of the ostial segment, leaving free space between vessel wall and stent (red arrows). B- Crushing procedure results in stent distortion (red pointed line). C- Coronary guiding wire tracking is unpredictable, it can follow the wrong path in the space between vessel wall and stent (purple and red interrupted lines) or the correct path through the space that allows its passage inside the lumen of the stent - Space of Optimal Wiring (SOW), (green line).

Figure 2. Micro-computed tomographic imaging of bench stents deployments using Crush technique. Coronary wire tracking in the wrong paths results in further SB stent distortion by the following steps of the procedure, that finally causes gaps in vessel coverage by stent at the end of the procedure (arrow-Gap in SB stent).

Figure 3. Proximal Side Optimization. Upper panel – graphic representation, lower panel-microphotography on the silicone phantom model. A- coronary bifurcation lesion involving

side branch. B- stent deployment in SB. C- the first step of Proximal Side Optimization (PSO)- the delivery balloon is pulled half-way back into the Main Branch (MB) and a second balloon inflation is performed at a pressure higher than the pressure of deployment. D- second step of the PSO procedure- Non Compliant balloon (NC), having a diameter +0.5 mm larger than the implanted stent, is delivered to the proximal segment of the stent and high pressure dilatation is performed. E- SB stent is crushed by a NC balloon in the MB.

Figure 4. The concept of Space of Optimal Wiring (SOW). A and D- Angiography “stent boost” imaging after crushing the SB stent that was implanted in PSO modification. The yellow pointed line shows the SOW, the white pointed line shows the crushed segment of the SB stent. B and E- graphic representation showing the concept of SOW (E- en face). C and F- IVUS imaging showing SB stent fully deployed after PSO before crush, and F- large SOW after Crush. Note that after application of PSO, wire tracking has no other possible passages except in the lumen of the stent.

Figures

Figure 1.

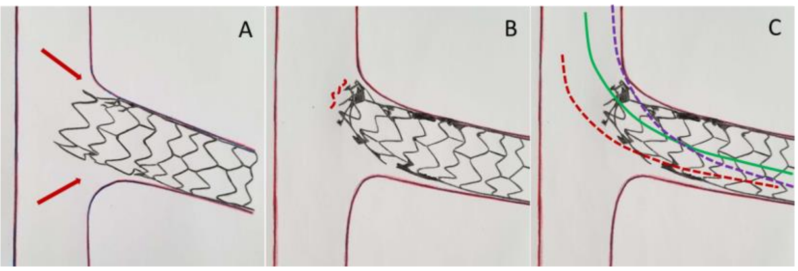

Figure 2.

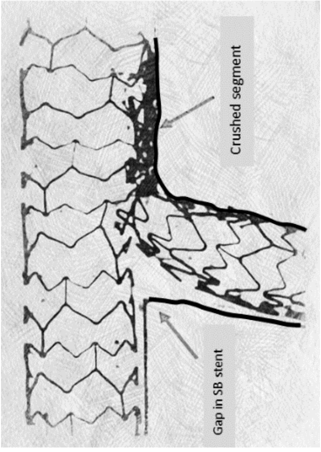

Figure 3.

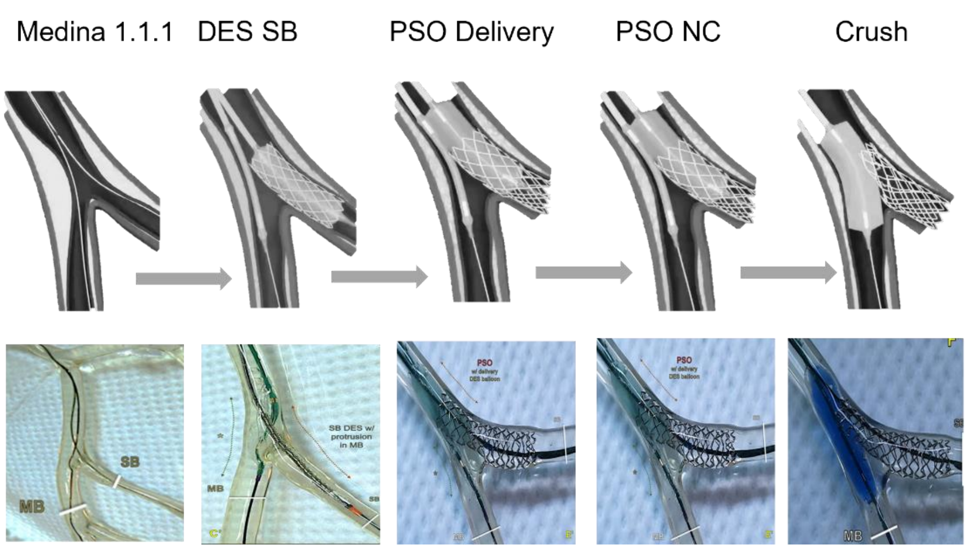

Figure 4.

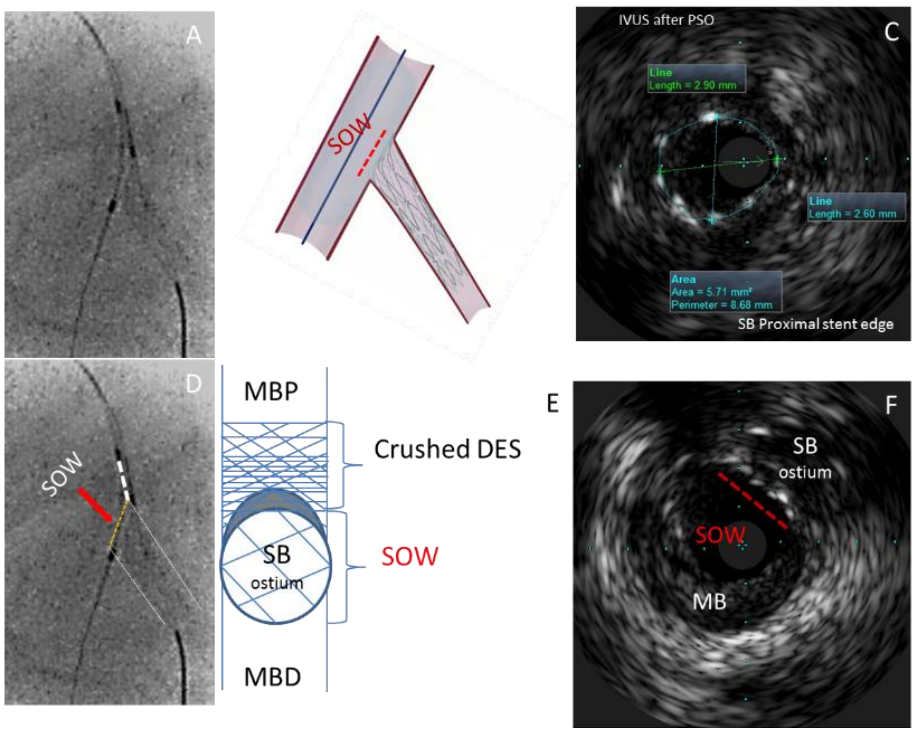

Supplement: Supplementary file 1 [file Data_Sheet_1.PDF]
